# The Association Between Obesity, Chronic Inflammation, Metabolic Disorders and Mood Disorders Among Patients up to 12 Months After Hospitalization for SARS-CoV-2

**DOI:** 10.3390/diagnostics14212357

**Published:** 2024-10-23

**Authors:** Kamila Rachubińska, Alicja Mińko, Iwona Rotter, Joanna Sołek-Pastuszka, Przemysław Ustianowski, Karolina Skonieczna-Żydecka, Elżbieta Grochans

**Affiliations:** 1 Department of Nursing, Pomeranian Medical University in Szczecin, Żołnierska 48, 71-210 Szczecin, Poland; kamila.rachubinska@pum.edu.pl (K.R.); przemyslaw.ustianowski@pum.edu.pl (P.U.); elzbieta.grochans@pum.edu.pl (E.G.); 2 Department of Medical Rehabilitation and Clinical Physiotherapy, Pomeranian Medical University, 71-210 Szczecin, Poland; iwrot@wp.pl; 3 Anesthesiology and Intensive Care, University Hospital No. 1 Unii Lubelskiej, 71-252 Szczecin, Poland; joanna.solek.pastuszka@pum.edu.pl; 4 Department of Biochemical Science, Pomeranian Medical University in Szczecin, Broniewskiego 24, 71-460 Szczecin, Poland; karolina.skonieczna.zydecka@pum.edu.pl

**Keywords:** anxiety, BMI, CBC, COVID-19, CRP, depression, glycemia, obesity, chronic inflammation

## Abstract

Background/Objectives: The relationship between BMI, inflammation, and mental health is complex. A high BMI, especially obesity, is associated with chronic inflammation, which can lead to mental disorders such as depression. Inflammatory cytokines affect neurotransmitters and the stress axis, worsening mental health. Obesity and mental disorders can mutually reinforce each other. New findings show that inflammation can lead to neurobiological changes, and the gut microbiota may play a key role. Obesity has been implicated as a factor in the high mortality and duration of influenza-like illnesses, even in people who do not have other chronic diseases that may increase the risk of complications. The aim of this study was to determine the associations between BMI and chronic inflammation, metabolic disorders, depression, and anxiety in patients hospitalized with COVID-19 up to 12 months after hospitalization. Methods: The study included 248 participants previously hospitalized for SARS-CoV-2 infection up to 12 months after hospitalization. The study was conducted in a multistage design using a diagnostic survey, anthropometric measurements, and laboratory methods. Results: A statistically significantly higher BDI-II score was observed among women. Statistical analysis showed a statistically significant higher GAD-7 score among women and those over 75 years of age. Conclusions: Higher BMI among subjects is often associated with elevated values of inflammatory markers and immune cells, such as WBC, neutrophils, monocytes, and CRP, as well as higher blood glucose levels. These associations may be related to the chronic inflammation and metabolic disorders that often accompany obesity. Lymphocytes and eosinophils may show more varied relationships depending on individual factors and specific health conditions. It is therefore important to continue research in this area.

## 1. Introduction

The COVID-19 pandemic significantly disrupted daily life worldwide. Government restrictions limited movement and led to the closure of many places, such as recreational facilities, gyms, and sports centers. As a result, people were forced to stay at home, leading to significant lifestyle changes, such as decreased physical activity, increased sedentary behavior, and elevated stress levels. Research shows that lockdowns contributed to reduced physical activity and worsened well-being. Abate Daga et al. [1] indicated that these restrictions negatively impacted subjective health, which is also confirmed by the findings of Franco et al. [2], who observed a deterioration in mental health related to limited physical activity.

Obesity can cause many physical disabilities and psychological problems, dramatically increasing the risk of developing many non-communicable diseases, including cardiovascular disease, cancer, and diabetes. In addition, it is often associated with a high number of hospitalizations and intensive care unit (ICU) admissions, with morbidity and mortality rates higher than population averages [3,4]. Obesity has been implicated as a factor in the high mortality and duration of influenza-like illnesses, even in people who do not have other chronic diseases that may increase the risk of complications [5]. As a chronic and multifactorial disease, obesity is associated with chronic inflammation that can cause a number of previously known and documented complications [6,7]. Studies have confirmed that high levels of pro-inflammatory cytokines produced in adipose tissue (mainly in men) and a ‘cytokine storm’, or over-activation of the immune system, may be possible causes of respiratory failure in the most severe forms of coronavirus disease 19 (COVID-19) [5]. Chronic diseases that often coexist with obesity, such as type 2 diabetes and hypertension, may be risk factors for severe forms of COVID-19 [8,9]. Obese patients often have physiological respiratory dysfunction, and, in addition, obesity increases the risk of hypoventilation-associated pneumonia, pulmonary hypertension, and cardiac stress, factors that may lead to a worse prognosis for COVID-19 [10]. Severe obesity is associated with sleep apnea syndrome, surfactant dysfunction, and inadequate glycemic control (which is associated with impaired ventilatory function) [11]. All of the aforementioned complications negatively affect the quality of life, well-being, and recovery period.

There is a large body of evidence showing an increase in the prevalence of psychiatric disorders worldwide during the COVID-19 pandemic, particularly the severity of anxiety and depression symptoms in both general populations and across age and occupational groups [12]. According to the results of a meta-analysis comparing anxiety levels among different populations, the group most at risk of developing anxiety disorders are patients hospitalized for COVID-19, with more than 43% having anxiety symptoms, compared to 29% among healthcare workers [13]. Most studies describing the impact of the COVID-19 pandemic on mental health have been conducted on the general population. Reports focusing on the mental health of COVID-19 patients are in the minority. A study by Janiri et al. found that approximately 30% of patients affected by COVID-19 had symptoms of post-traumatic stress disorder (PTSD), with a higher prevalence in women [14]. Another study found anxiety symptoms in more than one-third of patients hospitalized with COVID-19, with significantly higher rates in women, those over 50 years of age, those with lower levels of education, and those with a family history of COVID-19 infection [15]. The results from another study showed that patients hospitalized for SARS-CoV-2 infection were more likely to have symptoms of increased anxiety [16], and older age was an additional risk factor for increased depressive symptoms [17].

Due to the wide clinical spectrum, the mechanism of complications associated with the course of COVID-19 remains unclear; only a thorough understanding of the pathophysiology will allow the side effects of the disease to be assessed and treated [18,19]. Although the persistence of respiratory symptoms, particularly dyspnea and cough, appears to be common in Long-COVID, some persistent symptoms may be part of a multi-organ disease, and multidisciplinary management of these chronic sequelae is required [20]. A number of clinical features have been associated with mortality in patients infected with SARS-CoV-2, including older age, male gender, and obesity [21]. Among complete blood count (CBC) parameters, increased white blood cell (WBC) count, thrombocytopenia, lymphopenia, neutrophilia, and eosinopenia have been identified as markers of severity [21,22].

The aim of this study was to determine the associations between BMI and chronic inflammation, metabolic disorders, depression, and anxiety in patients hospitalized with COVID-19 up to 12 months after hospitalization. The study presented here is intended to help assess the need for psychological support and to enable the selection of variables influenced by the pandemic phenomenon.

## 2. Materials and Methods

We conducted the study described in this paper between 1 December 2021 and 31 October 2022 in Szczecin, Poland. The study sample consisted of 248 participants, all previously hospitalized for SARS-CoV-2 infection. To qualify for the study, participants had to be aged 18 years or older, have had a confirmed hospital stay due to COVID-19 (confirmed by PCR test and CT scan), and have had no history of diagnosed mental illness or metabolic disorders (e.g., stroke, cancer, or autoimmune diseases) within two months before enrollment (inclusion criteria). Other exclusion criteria included prior liver or renal failure, vascular diseases, or thyroid conditions. Informed consent was obtained from all participants before the study. Every effort was made to protect patient privacy and anonymity. The study was conducted in accordance with the current version of the Declaration of Helsinki. Consent to conduct was obtained from the Bioethics Committee of the Pomeranian Medical University in Szczecin, Resolution no. KB-0012/161/2021 dated 31 May 2021.

### 2.1. Study Design

Following approval of the survey, the survey sheets were handed over in person by a trained interviewer to participants who had read the above information and agreed to participate in the survey. Participants were informed about the purpose of the study, the anonymity of their responses, and the lack of access by outsiders to their data. Those qualified for the study were able to ask questions, remain anonymous, and receive comprehensive explanations. This study had a multistage design using a diagnostic questionnaire, anthropometric measurements, and laboratory methods. It identified dependent variables (prevalence of anxiety and depressive disorders) and independent variables (sociodemographic factors, namely age, gender, education, place of residence, marital status, work activity, and BMI, as well as qualitative and quantitative assessment of morphotic elements, glucose, and CRP).

### 2.2. Surveys

This study was carried out by means of a diagnostic survey using a questionnaire technique. Standardized tools adapted to Polish conditions were used.

The Generalized Anxiety Disorder Questionnaire (GAD-7, Generalized Anxiety Disorder) is a screening tool used to determine feelings associated with generalized anxiety syndrome. The questionnaire consists of 7 questions. Each question has a score from 1 to 3, the sum of which indicates the severity of the anxiety: 0–4 (no anxiety), 5–9 (mild anxiety), 10–14 (moderate anxiety), and 15–21 (severe anxiety). The GAD7 version was translated into Polish by the MAPI Research Institute. Considering the results, the Cronbach’s alpha reliability coefficient for the questionnaire in our study was α = 0.94 [23,24].

The Beck Depression Inventory (BDI I-II) is used to self-assess the severity of depressive disorders. It contains 21 questions with four response options. The final score is calculated by the sum of the scores obtained for each question. They reflect the level of depression and are interpreted as follows:

- 0–13—no depression or minimal depressive symptoms;
- 14–19—mild depression;
- 20–28—moderate depression;
- 29–63—severe depression.

The BDI-II scale showed adequate internal consistency before and after treatment (α = 0.87 and 0.90, respectively) [25,26].

The self-administered survey questionnaire included closed and semi-open questions to obtain selected sociodemographic data (age, education level, marital status, place of residence, and work activity).

### 2.3. Laboratory Tests

Blood was drawn from the ulnar vein of each study participant after overnight fasting on the following day between 7.00 and 9.30 a.m. following a 10-min rest in a sitting position, using a Vacutainer system (BD Vacutainer Eclipse, BD). Blood was collected by qualified nurses in accordance with current policies and procedures for the collection, storage, and transport of biological material. Plasma was prepared from blood by centrifugation, frozen in portions, and stored at −80 °C until laboratory analysis. Determinations of biochemical parameters were performed in a certified laboratory of the Pomeranian Medical University in Szczecin using standardized commercial methods. Blood was collected for qualitative and quantitative assessment of morphotic elements, glucose, and CRP.

### 2.4. Anthropometric Measurements

In the next stage of the study, anthropometric measurements were taken as follows:

Body weight and height were assessed using a certified medical scale together with an integrated SECA 711 growth meter according to a standard procedure to the nearest 0.1 kg of body weight and 0.1 cm of height, respectively. Participants stood with their backs straight, heels joined together, barefoot and lightly dressed.

BMI (body mass index) was calculated using the formula BMI = weight (kg)/height (m)^2^ = BMI (kg/m^2^) and divided into the following categories according to World Health Organization (WHO) criteria:

- Underweight (BMI < 18.5 kg/m^2^);
- Normal weight (BMI = 18.5–24.9 kg/m^2^);
- Overweight (BMI = 25.0–29.9 kg/m^2^);
- Grade 1 obesity (BMI = 30.0–34.99 kg/m^2^);
- Grade 2 obesity (BMI = 35.0–39.99 kg/m^2^);
- Grade 3 obesity (BMI = over 40 kg/m^2^) [26].

### 2.5. The Statistical Analysis

Statistical analysis was performed using Statistica 13.1 software (Stat Soft, Inc., Tulsa, OK, USA). Descriptive statistics including the number of patients, percentage of patients, mean, median, standard deviation, and interquartile ranges were used to characterize the study group. The normality of distribution was assessed using the Shapiro–Wilk test. The Mann–Whitney U test was used to analyze the differences between individual groups, while the Kruskal–Wallis test was used to analyze differences between multiple groups. Statistical significance was attributed to results for which the *p*-value was less than 0.05.

## 3. Results

There were 248 participants in the study, the majority of whom were women (56.3%). The mean age of the study group was 60.51 years. More than one-third of the subjects were overweight (36.2%), and more than one-fourth had Grade 1 obesity according to the WHO criteria (25.4%). Table 1 shows the detailed characteristics of the study group. 

Due to the comprehensiveness of the analyses carried out, and for the sake of the clarity of the research results presented in the article, the following is limited to the presentation of statistically significant results, and analyses of variables from the BMI subgroup were carried out based on the following breakdown criteria:

- Underweight and normal weight: <25;
- Overweight: ≥25 < 30;
- Obesity: ≥30 (30.0–34.99 kg/m^2^ (Grade 1 obesity); 35.0–39.99 kg/m^2^ (Grade 2 obesity); over 40 kg/m^2^ (Grade 3 obesity)).

Analysis of the data showed that among the CBCs, platelet distribution width (PDW) (12.4495 fL vs. 6.1–11.0 fL) and mean platelet volume (MPV) (10.6081 fL vs. 7.5–10.5 fL) values were statistically significantly above normal. Among the study group, higher blood glucose levels were also demonstrated compared to normal levels (105.5725 mg/dL vs. 70–99 mg/dL). Table 2 presents the descriptive characteristics of the laboratory parameters of the study group along with the normal range.

A statistically significantly higher BDI-II score was observed among women (M = 12.29; *p* = 0.023). There were no statistically significant associations between depression according to the BDI I-II and BMI or age (*p* < 0.005) (Table 3).

Statistical analysis showed a statistically significant higher GAD-7 score among women (M = 7.54; *p* = 0.022) and those over 75 years of age (M = 9.58; *p* = 0.045). No association was found between anxiety according to GAD-7 and BMI (*p* = 0.611) (Table 4).

Table 5 presents data on the comparison of selected laboratory parameters in relation to BMI. Additional multiple-comparison analysis was performed for statistically significant results. A statistically significant relationship was found between the group with BMI < 25 and the group with BMI ≥ 30 and the levels of leukocytes (*p* < 0.001), neutrophils (*p* = 0.005), lymphocytes (*p* < 0.001), monocytes (*p* = 0.001), glucose (*p* < 0.001), and CRP (*p* = 0.006). There was a statistically significant relationship between the group with BMI ≥ 25 and BMI < 30 and the group with BMI ≥ 30 with levels of leukocytes (*p* < 0.001), neutrophils (*p* = 0.013), lymphocytes (*p* = 0.032), and CRP (*p* = 0.001).

## 4. Discussion

The COVID-19 pandemic significantly affected the relationships between BMI and various hematological, biochemical, and psychological indices in people hospitalized with the disease. The results obtained in this study, which assessed the occurrence of BMI and selected laboratory parameters (morphology, glucose, and C-reactive protein) and psychosocial functioning (anxiety and depression) of patients hospitalized for COVID-19 up to 12 months after hospitalization, contribute to the literature in several ways, as explained below. The statistical analysis results are for comparing groups.

Obesity is a recognized independent predictor of severe H1N1 infection [27,28]. A higher BMI is considered a potential risk factor for serious complications in hospitalized patients with COVID-19 infection [29,30,31,32,33]. The study by Docherty et al. [34], conducted on a group of over 20,000 patients hospitalized due to COVID-19 in the USA, found that individuals with obesity (BMI ≥ 30) had a 48% higher risk of hospitalization and a 74% higher risk of admission to the intensive care unit (ICU) compared to those with normal body weight. The risk of death was 36% higher in individuals with obesity. Additionally, in the study by Hamer et al. [35], which included a retrospective analysis of treatment outcomes for 5700 patients from New York, it was noted that individuals with a BMI > 35 had a 3.6 times higher risk of requiring mechanical ventilation and a 1.5 times higher risk of death. According to the results of our study, almost half of the subjects were obese, and more than one-third were overweight. For hospital cohorts, the prevalence of obesity and overweight was high in patients with COVID-19 infection and was associated with the worst prognosis [36,37]. Interestingly, overweight/obesity was associated with the likelihood of prolonged hospitalization in patients with COVID-19 infection, highlighting the role of high BMI as a predictor of poorer prognosis in hospitalized patients. The available studies showed that obese patients were more likely to need hospitalization than non-obese patients. In addition, obese patients had a more severe course, requiring home low-flow oxygen therapy, NIV, and intubation. Death from COVID-19 was also higher in obese patients compared to non-obese patients [38].

Numerous studies indicate that obesity increases the risk of severe COVID-19. This is associated with chronic inflammation, immune dysfunction, and metabolic abnormalities, which are often seen in patients with higher BMI. Patients with obesity are more likely to require hospitalization, intensive care, and mechanical ventilation due to more severe COVID-19 symptoms. Higher BMI is associated with poorer clinical outcomes, including an increased risk of death [39,40].

### 4.1. BMI and Anxiety and Depression

Anxiety and depressive symptoms may be more common in people following SARS-CoV-2 infection, and several reports suggest that COVID-19 patients are at increased risk of mood and anxiety disorders 3 months after infection [40,41,42,43].

Depression and anxiety are the most common mental health conditions, often bringing severe internal pain to sufferers and placing a severe burden on their families. During the SARS-CoV-2 pandemic, people all over the world had to change their lifestyles, and most were forced to stay at home or significantly reduce outdoor activities, which could harm their mental well-being [6,7]. According to the analysis of our study, more than half of the subjects showed anxiety and depressive symptoms up to 12 months after their hospitalization. Scores were high despite the passage of time.

Evidence suggests a positive association between increased BMI and psychiatric conditions such as depression, anxiety, mania, panic attacks, social phobia, and suicidal thoughts [43]. In contrast, our own study showed no relationship between anxiety, depressive symptoms, and BMI, which may be related to the fact that more than three-quarters of the group were overweight or obese, and the overall prevalence of anxiety and depressive symptoms across the group was very high. Being overweight or obese may be a risk factor for poor mental health, which may also result in higher suicide rates. In a study by Auny et al. [44], subjects with a higher BMI were significantly more prone to depression and anxiety, compared to those with a normal BMI. As mentioned above, changes in BMI increase the risk of depressive and anxiety disorders.

Depression and anxiety can weaken the immune system, which may increase susceptibility to infection and worsen the course of COVID-19, particularly among those hospitalized for COVID-19. People who are obese and also experience depression may have an increased risk of complications. A negative mental state can lead to eating disorders such as emotional eating, which in turn can lead to further weight gain and deterioration of physical and mental health. Hospitalization for COVID-19 affected the relationships between BMI and various hematological and biochemical indices in a way that highlights pre-existing obesity-related health problems and introduces new challenges related to the body’s response to viral infection [44].

### 4.2. Relationship Between BMI and Glycemic Levels 

The results of our study showed that blood glucose levels among the study group were high, averaging 105 mg/dL. Higher BMI is strongly associated with higher blood glucose levels. This relationship is due to the insulin resistance often found in people with higher BMI, leading to conditions such as pre-diabetes and type 2 diabetes [38]. Obesity is a risk factor for insulin resistance and type 2 diabetes, leading to elevated blood glucose levels. COVID-19 infection can lead to hyperglycemia, especially in patients with a higher BMI. Patients with obesity and COVID-19 often present with elevated blood glucose levels as a result of insulin resistance and metabolic stress caused by the infection. Hyperglycemia is associated with poorer clinical outcomes and can lead to complications such as type 2 diabetes [38,39].

### 4.3. Relationship Between BMI and CBC

The relationships between body mass index (BMI) and various blood parameters, such as white blood cell (WBC) counts, neutrophils, lymphocytes, monocytes, eosinophils, glucose levels (GLU), and C-reactive protein (CRP), can be complex and vary depending on the clinical context and the patient’s health status. Our study showed that among patients hospitalized for up to 12 months with COVID-19, higher values were seen in the group with BMI > 25 regarding WBC, neutrophil, lymphocyte, monocyte, and eosinophil count. A higher BMI is associated with increased WBC, neutrophile, and monocyte levels. These values are associated with the severity and presence of conditions such as carotid atherosclerotic plaques, suggesting that inflammation and immune response are increased with a higher percentage of body fat [45,46]. COVID-19 infection often leads to elevated neutrophile and monocyte counts, and people with obesity may have even higher values due to existing inflammation. Increased WBC counts are associated with a more intense inflammatory response, which can worsen patients’ condition. Lymphocyte counts tend to decrease with higher BMI, while eosinophile counts may show a varied response depending on specific conditions and demographic factors. Lymphopenia, a reduction in lymphocyte count, is a common finding in patients with COVID-19. Those with a higher BMI may have more pronounced changes, suggesting immune dysfunction in this group. COVID-19 may affect monocyte and eosinophile counts, although these changes are more variable and may depend on the severity of the infection and the presence of other comorbidities [38,39].

### 4.4. Relationship Between BMI and CRP

CRP, as a marker of inflammation, tends to be elevated in patients with COVID-19. Our own research has shown that among patients hospitalized for up to 12 months with COVID-19, being overweight is associated with elevated CRP levels, compared to normal weight and obesity. It is interesting that CRP levels are higher in overweight than in obese individuals. Further research is needed on this topic. By contrast, the results of other authors indicate obesity further increases CRP levels, which may indicate chronic inflammation. A high CRP level is associated with poorer clinical outcomes, including increased risk of complications and death [46].

There are serious concerns about the impact of COVID-19 on mental health. The pandemic has caused many stressors (health, economic, social, etc.) that individually and collectively harm mental health. However, the effects on mental health are unlikely to be the same across the population; some people are likely to be at greater risk than others. This study indicates that higher BMI is a pre-existing vulnerability to mental health deterioration. It has been found to consistently lead to poor mental health [47] and physical health outcomes [48]. In particular, previous research has shown that being overweight and obese is associated with both concurrent depressive symptoms and an increase in depressive symptoms over time [49]. In the study by Plakhotnik et al. [50], conducted in South Africa on a group of 400 COVID-19 patients, individuals with a BMI > 30 had a 2.5 times higher risk of severe disease and a 3 times higher risk of requiring mechanical ventilation. Obesity was also associated with higher levels of inflammatory markers, such as CRP and interleukin-6 (IL-6), indicating an increased inflammatory state.

COVID-19 can significantly modify the relationships between BMI and various health indicators, especially in the context of inflammation, immune response, and metabolism. Individuals with a higher BMI are more likely to have a severe disease course, which is associated with pre-existing metabolic and immune abnormalities. COVID-19 clearly highlights and exacerbates pre-existing obesity-related health problems. Chronic inflammation, immune dysfunction, and metabolic abnormalities, which are common in people with higher BMI, can lead to a more severe disease course and increased risk of hospitalization and complications. SARS-CoV-2 infection puts additional stress on the body, resulting in more pronounced changes in hematological and biochemical parameters such as WBC, neutrophils, lymphocytes, monocytes, eosinophils, glucose levels, and CRP. People with a higher BMI are more likely to develop mental health problems such as depression and anxiety. Chronic inflammation and endocrine dysfunction associated with obesity may contribute to worsened mood and increased vulnerability to mental health disorders.

Worldwide, obesity has proven to be a key risk factor for severe COVID-19, as confirmed by numerous studies from various regions. The pandemic has revealed how the global issue of obesity can lead to exacerbated symptoms of viral infections. Each region has differences in obesity rates and comorbidities, which affect specific study outcomes, but the overall trend indicates that a higher BMI significantly worsens health outcomes for COVID-19 patients. These findings highlight the necessity of a global fight against obesity and the implementation of individualized therapeutic approaches in different populations. The global context indicates that obesity not only worsens physical health outcomes in COVID-19 patients but also affects their mental health. This underscores the need for further research into the mechanisms linking obesity, inflammation, and mental health in various populations [51,52,53,54,55,56].

### 4.5. Limitations and Strengths

The discrepancies presented in this study have identified some limitations and implications for professional practice. The strengths of this study include a well-defined population of patients following hospitalization for COVID-19 with comprehensive information, including hospital and personal data. However, this study also has some limitations. First, although SARS-CoV-2 infection is the exclusive focus of this study, it was revealed that other viral infections, such as seasonal influenza, may have long-term effects similar to those following SARS-CoV-2 infection. Therefore, it is uncertain whether our findings are specific to this particular viral infectious disease or whether they point to post-viral syndromes more broadly. An additional difficulty was the lack of values for several variables of interest. Furthermore, the lack of data on the parameters studied prior to infection makes it impossible to assess changes. The study presented here also has limitations regarding the lack of data on the variables studied before the pandemic period, which could be relevant to the current level of these variables. Another limitation is that the results are inconclusive as to whether they are due to post-COVID infection or obesity itself. This study could be strengthened by comparing the results after the infection phase subsided and again 12 months later. Despite the limitations, the present study provides important findings and may be a starting point for a larger study on the nutritional, psychosocial, and selected laboratory parameters of patients hospitalized with COVID-19.

## 5. Conclusions

A higher BMI among subjects is often associated with elevated values of inflammatory markers and immune cells, such as WBCs, neutrophils, and monocytes, as well as higher blood glucose levels. These associations may be related to the chronic inflammation and metabolic disorders that often accompany obesity. Lymphocytes and eosinophils may show more varied relationships depending on individual factors and specific health conditions. It is interesting that CRP levels are higher in overweight than in obese individuals. It is therefore important to continue research in this area.

## Figures and Tables

**Table 1:** Characteristics of the study group.

Variable		*n*	%
Gender	Female	140	56.3
	Male	108	43.7
Age	<50 years	58	23.3
	≥50 < 75 years	154	61.9
	≥75 years	36	14.8
Nutritional status (BMI)	<18.5 kg/m^2^ (underweight)	0	0.0
	18.5–24.99 kg/m^2^ (normal weight)	59	23.7
	25.0–29.9 kg/m^2^ (overweight)	91	36.5
	30.0–34.99 kg/m^2^ (Grade 1 obesity)	63	25.5
	35.0–39.99 kg/m^2^ (Grade 2 obesity)	27	10.9
	over 40 kg/m^2^ (Grade 3 degree obesity)	8	3.4
BDI-I-II depressive disorders	Lack of depressiveness	142	57.3
	Depressiveness	106	42.7
Anxiety disorders according to GAD-7	No symptoms of anxiety	101	40.7
	Anxiety symptoms	147	59.3

Legend: *n*—number of subjects; BMI—body mass index; GAD-7—General Anxiety Disorder-7; BDI I-II—The Beck Depression Inventory.

**Table 2:** Descriptive characteristics of selected laboratory parameters.

Laboratory Parameters (Unit)	Standard	M	Me	Min–Max	IQR	SD
WBC (thousands/µL)	4–10	6.0788	5.740	1.240–12.120	2.290	1.84988
RBC (million/µL)	4.5–5.5	4.6402	4.650	2.810–5.730	0.590	0.49531
Hemoglobin (g/dL)	12–15.5	13.7763	13.900	9.500–17.500	1.800	1.38855
Hematocrit (%)	40–51	40.2722	40.600	29.100–48.800	4.000	3.66278
MCV (fL)	81–99	87.0361	86.800	74.200–103.600	4.400	4.14606
MCH (pg)	27–32	29.7629	29.600	24.000–36.700	1.700	1.72085
MCHC (g/dL)	32–36	34.1959	34.100	31.900–36.400	1.600	1.03591
PLT (thousands/µL)	180–430	251.5567	243.000	67.000–543.000	84.000	69.04663
PDW (fL)	6.1–11	12.4495	12.300	8.700–20.100	2.500	2.13445
MPV (fL)	7.5–10.5	10.6081	10.600	0.000–43.700	1.400	2.63055
Neutrophils (%)	40–75	54.9583	54.700	15.100–92.3000	14.300	10.98555
Lymphocytes (%)	25–45	32.2685	31.850	3.500–78.200	12.300	10.27382
Monocytes (%)	2–12	9.3879	8.700	1.500–113.100	2.600	7.68633
Eosinophils (%)	1–6	2.8685	2.600	0.000–9.200	2.000	1.69177
Basophils (%)	0–1	0.6182	0.600	0.000–1.700	0.400	0.35284
GLU (mg/dL)	70–99	105.5725	96.000	79.600–344.000	16.400	33.77921
CRP (mg/L)	<5	4.4410	1.620	0.000–131.590	3.140	11.35036

Legend: M—mean; Me—median; Min—minimum value; Max—maximum value; IQR—interquartile range; SD—standard deviation; WBC—white blood cell; RBC—red blood cell; MCV—mean corpuscular volume; MCH—mean corpuscular hemoglobin; MCHC—mean corpuscular hemoglobin concentration; PLT—platelets; PDW—platelet distribution width; MPV—mean platelet volume; GLU—glucose; CRP—C-reactive protein.

**Table 3:** Associations between depression according to BDI I-II and gender, age, and BMI.

Variable		M (SD)	*p*
Sex	Woman	12.29 (8.89)	0.023 *^
	Man	10.04 (8.67)	
BMI (kg/m^2^)	<25 kg/m^2^	12.08 (8.38)	0.538 ’
	≥25 < 30 kg/m^2^	10.96 (8.72)	
	≥30 kg/m^2^	11.36 (9.26)	
Age (Years)	<50	10.43 (7.66)	0.112 ’
	≥50 < 75	11.16 (9.37)	
	≥75	13.59 (8.29)	

Legend: M—mean; SD—standard deviation; BMI—body mass index; *p*—statistical significance, * *p* < 0.05; ^—Mann–Whitney U test; ’—Kruskal–Wallis test.

**Table 4:** Associations between anxiety according to GAD-7 and gender, age, and BMI.

Variable		M (SD)	*p*
Sex	Woman	7.54 (6.02)	0.022 *^
	Man	5.89 (5.71)	
BMI (kg/m^2^)	<25	7.34 (5.86)	0.611 ’
	≥25 < 30	6.76 (5.93)	
	≥30	6.67 (6.04)	
Age (years)	<50	6.69 (5.57)	0.045 *’
	≥50 < 75	6.22 (5.52)	
	≥75	9.58 (7.34)	

Legend: M—mean; SD—standard deviation; BMI—body mass index; *p*—statistical significance, * *p* < 0.05; ^—Mann–Whitney U test; ’—Kruskal–Wallis test.

**Table 5:** Comparison of selected laboratory parameters in relation to BMI.

Variable (Unit)	BMI < 25 kg/m^2^	BMI ≥ 25 and BMI < 30 kg/m^2^	BMI ≥ 30 kg/m^2^	*p*
	M (±SD)	M (±SD)	M (±SD)	
WBC (thousands/µL)	5.11 (1.60)	5.63 (1.62)	7.24 (1.68)	<0.001 *’
RBC (million/µL)	4.57 (0.59)	4.60 (0.51)	4.75 (0.39)	0.458 ’
Hemoglobin (g/dL)	13.55 (1.48)	13.62 (1.49)	14.16 (1.12)	0.252 ’
Neutrophils (thousands/µL)	3.46 (1.76)	3.65 (1.93)	4.11 (1.46)	0.002 *’
Lymphocytes (thousands/µL)	1.77 (0.56)	2.04 (0.78)	2.32 (1.18)	<0.001 *’
Monocytes (thousands/µL)	0.51 (0.20)	0.58 (0.21)	0.62 (0.17)	0.002 *’
Eosinophils (thousands/µL)	0.16 (0.11)	0.17 (0.11)	0.21 (0.12)	0.028 *’
Basophils (thousands/µL)	0.03 (0.02)	0.04 (0.02)	0.04 (0.02)	0.777 ’
GLU (mg/dL)	98.4 (29.52)	102.08 (29.51)	113.41 (40.54)	<0.001 *’
CRP (mg/L)	4.23 (8.13)	5.35 (8.13)	3.73 (3.72)	0.004 *’

Legend: M—mean; SD—standard deviation; BMI—body mass index; WBC—white blood cell; RBC—red blood cell; GLU—glucose; CRP—C-reactive protein; *p*—statistical significance, * *p* < 0.05; ’—Kruskal–Wallis test.

## Data Availability

The data that support the findings of this study are available from the corresponding author (A.M.) upon reasonable request. The data are not publicly available due to the principle of anonymity of the people who took part in the study. The participants signed a data confidentiality clause.
